# Association between metabolic syndrome and work: an integrative review of the literature

**DOI:** 10.47626/1679-4435-2020-511

**Published:** 2020-12-11

**Authors:** Amália Ivine Costa Santana, Magno Conceição das Merces, Lucélia Batista Neves Cunha Magalhães, André Luiz Brandão Costa, Argemiro D'Oliveira

**Affiliations:** 1 Programa de Pós-Graduação em Ciências da Saúde, Universidade Federal da Bahia (UFBA) - Salvador (BA), Brazil; 2 Departamento de Ciências da Vida, Universidade do Estado da Bahia - Salvador (BA), Brazil; 3 Medicina, UFBA - Salvador (BA), Brasil

**Keywords:** metabolic syndrome, occupational health, occupational risks

## Abstract

The aim of this study was to highlight the association between occupational aspects and the occurrence of metabolic syndrome among various occupational groups. This is an integrative review of the literature that included articles indexed in the following databases: LILACS, SciELO, PubMed, and CINAHL. We evaluated 32 articles, most of which were published in Englishlanguage medical journals and with level 4 scientific evidence. The occupational aspects most commonly reported as associated with metabolic syndrome were occupation, work shift, and occupational stress. Our results indicated that occupational aspects could negatively interfere with workers’ health; more robust longitudinal studies should contribute to further uncovering the reported associations.

## INTRODUCTION

Work is the most genuine form of building relationships and one’s own story; the importance of work on people’s lives has been consistently reported by the scientific literature.^1^ In addition to providing means of living, work helps defining the status of an individual within the society and his or her own personal identity; it allows the development of time management skills and the enrichment of social relations.^2^

The labor process takes place when the human being transforms nature in order to attribute a purpose to it; labor is thus connected to a specific objective and is concluded when the product is finished. The product is a use value, suitable for satisfying a certain need.^3^

Considering all the contributions of work to human life, it should only be a source of pleasure. However, work can have negative effects on the human health, and the relationship between labor and the health-disease process is now well established. In this context, aspects of current work contribute to changes in neuroendocrine mechanisms, exposing workers to various risk factors that can ultimately lead to incapacity, disease, or even death.^4^

Among the problems faced by workers due to the execution of their work tasks, the metabolic syndrome (MS) is a clinical condition that aggregates various cardiovascular risk factors; these have prompted an increasing number of scientific publications on this condition. People with MS face a risk twice as high of cardiovascular diseases and one and a half higher for all-cause mortality in comparison to people without the syndrome. Within the Brazilian economically active population, the prevalence of MS has varied from 4.2% to 15.4%, according to a recent literature review.^5^^,^^6^

Many factors have been attributed as responsible for the development of MS: Insulin resistance, abdominal obesity, hypertension, and low levels of high-density lipoprotein (HDL) cholesterol are considered defining genetic factors; physical inactivity, age, pro-inflammatory state, and hormonal changes can also have determinant effects.^7^ Epidemiological studies have identified additional factors associated with the occurrence of MS, such as low schooling, social inequality, physical inactivity, dietary patterns, alcoholism, smoking habits, psychosocial tension, and work conditions.^7^^-^^11^

Studies that provide scientific evidence regarding the association between work and MS are scarce in the literature. Therefore, the aim of the present study was to highlight, based on the present literature, the association between occupational aspects and the occurrence of MS among various occupational groups.

## METHODS

This is an integrative review; this type of study summarizes the theoretical and empirical findings of previous investigations by using a rigorous evaluation method and with the aim of guiding clinical practice.^12^ The databases researched for this work were LILACS, SciELO, PubMed, and CINAHL.

Our sample included articles fully available online and published in Portuguese, English, and Spanish, with no publication date restrictions. We excluded abstracts published in annals, experience reports, editorials, and undergraduate, masters’, or Ph.D. theses/dissertations. Articles that did not clearly identify their methodological procedures were also removed from our review.

Data collection happened between May and June 2019, using the following guiding question: “is there an association between MS and work-related aspects?” We used a PICO strategy where the patient (P) aspect involved workers of any occupational category; intervention (I) represented work-related variables; comparison (C) considered the general population; and the outcome (O) aspect consisted of the occurrence of MS.

We used Portuguese, English, and Spanish keywords according to the Health Sciences Descriptors (DeCS) and their Medical Subject Headings (MeSH) counterparts: “metabolic syndrome,” “working conditions,” “work,” “working environment, ”occupational stress,” “occupational health,” and “occupational diseases.” Our search strategy combined 2 descriptors with a truncating technique using the following Boolean expressions: “metabolic syndrome” AND “working conditions”; “metabolic syndrome” AND “work”; “metabolic syndrome” AND “working environment”; “metabolic syndrome” AND “occupational stress”; “metabolic syndrome” AND “occupational health”; and “metabolic syndrome” AND “occupational diseases.”

Article pre-selection was performed by thoroughly reading titles and abstracts in order to verify the suitability of the subject according to inclusion criteria. Subsequently, a full-text reading of the pre-selected articles was performed, further excluding those that did not correspond to our criteria, as well as duplicate articles.

Finally, the selected articles were classified in levels of evidence according to the Agency for Healthcare Research and Quality (AHRQ). This classification divides levels of scientific articles into 7 levels of evidence: (1) systematic review or meta-analysis of randomized clinical trials; (2) randomized clinical trials; (3) non-randomized clinical trials; (4) cohort and case-control studies; (5) systematic review of descriptive and qualitative studies; (6) single descriptive or qualitative study; and (7) expert opinions or committee reports.^13^ According to our exclusion criteria, we did not considered levels of evidence 1, 5, or 7.

The analysis of the obtained data was performed in a descriptive manner in order answer the guiding question and considering the relevant ethical aspects for this type of study.

## RESULTS

**Figure 1:**
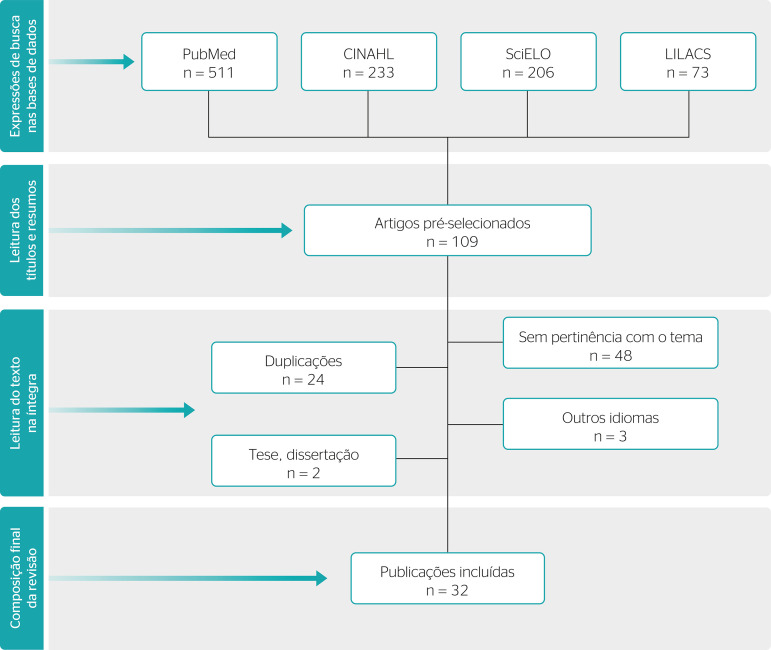
Article selection flowchart.

This literature review analyzed 32 articles. Figure 1 summarizes the methodology for database search and article selection. The platform that contributed with the most articles was PubMed (71.9% of the final publications), followed by SciELO (15.6%), LILACS (9.4%), and CINAHL (3.1%).

The selected articles were published from 2005 to 2018; the year with the most publications was 2015 (21.2%). Most of the selected studies were in English (78.8%), while 12.1% were in Portuguese and 9.1% were in Spanish.

Most of the studies were conducted in the Americas (39.4%) and Asia (39.4%); European publications represented 21.2% of the selected articles. Within the Americas, Brazil was the leading country in the number of studies (46.2%).

Most of the selected studies were cross-sectional (78.8%); cohort studies represented 21.2% of the selected articles, while case-control designs represented 3%. Therefore, studies with a level 6 of evidence were the most represented (75.8%), while level 4 studies accounted for 24.2%. We did not observe other AHRQ levels of evidence among the selected studies.

The area of knowledge with the most studies was medicine (45.5%), followed by occupational medicine (24.2%). Other identified areas of knowledge were public health (15.2%), nursing (12.1%), and mental health (3%).

An association between occupational aspects and MS has become evident in this review. The associations presented on Table 1 presented statistical significance. The analyzed aspects included occupational stress, work shift, duration of employment, working environment, occupation, weekly workload, employment type, occupational trauma, and justice at work. Risk factors for MS identified in this review included occupation (30.3%), work shift (27.2%), and occupational stress (15.1%).

**Table 1:** Selected studies and corresponding scientific journals, authors, countries of origin, publication dates, types, objectives, and conclusions (N = 32).

Journal	Authors	Country/ year	Objective	More Information
Revista Espanola de Cardiología	Alegría et al.^14^	Spain/ 2005	To evaluate the prevalence of MS among active Spanish workers and describe differences related to job types.	MS prevalence was 10.2%; it was higher among manual workers (11.8%) and lower among office (9.3%) and managerial (7.7%) workers.
British Medical Journal	Chandolaet al.^8^	England/ 2006	To investigate the associations between stress at work and MS.	Employees with chronic stress at work had twice as many changes of presenting MS than those who did not face this condition (RR = 2.25).
Diabetes Care	Davila et al.^15^	United States of America/2010	To evaluate differences in MS prevalence between different occupational groups.	The criteria for MS were met by 20% of workers.Transportation/material moving workers had higher odds of meeting the criteria when compared to executive, administrative, and managerial workers (OR = 1.70).
Medicina y Seguridad del Trabajo	Baldeon &Chumbes^16^	Peru/ 2010	To investigate the prevalence of MS among industrial and administrative workers.	The global prevalence of MS was 27.83%. Industrial workers had a MS prevalence of 28.94%, while administrative workers presented 23.07% of prevalence.
Ciência &Saúde Coletiva	Felipe-de-Melo et al.^17^	Brazil/ 2011	To identify factors associated with MS in administrative workers of an oil company.	MS was present in 15% of the workers. In higher-ranking jobs, prevalence was 12.4, while in the remaining positions, it was 16.4%.
Occupational and Environmental Medicine	Gimeno et al.^18^	England/ 2010	To evaluate if high levels of justice at work protect workers against MS.	Men that experienced high levels of justice at work presented lower risks of MS when compared to employees that faced low levels of justice (RR = 0.75).
International Journal of Emergency Mental Health and Human Resilience	Hartley et al.^19^	United States of America/ 2011	To examine the association between stress levels of police officers and MS and its individual components.	The general prevalence of MS was 26.7%. Work-related stress, organizational pressure, and lack of social support were associated with MS among female, but not male police officers (OR = 1.32).
Revista Portuguesa de Cardiologia	Rossa et al.^20^	Brazil/ 2012	To determine the prevalence of MS and variables related to its development in hospital workers.	MS diagnosis was confirmed in 12.8% of workers. Full- time work and employment duration > 10 years were associated with MS. There were no associations to occupational groups.
BMC Public Health	Kobayashi et al.^21^	Japan/ 2012	To investigate the relationship between long working hours and MS in Japanese workers.	MS was identified in 11.8% of workers, and there was an association between working 10 hours/day and MS (OR = 2.32).
International Journal of Occupational Medicine and Environmental Health	Mohebbi et al.^22^	Iran/ 2012	To evaluate the effect of shift work on the development of MS.	MS was more commonly diagnosed in people who working shifts (OR = 1.49).
Scandinavian Journal of Work, Environment & Health	Puttonen et al.^23^	Finland/ 2012	To evaluate if the risk of MS is increased in people working shifts.	Shift work was associated with a higher prevalence of MS (OR = 1.64).
Clinics	Salaroli et al.^24^	Brazil/ 2013	To determine the prevalence of MS in bank workers and to identify the related risk factors.	There was a higher prevalence of MS among individuals in lower-ranking jobs (OR = 2.6).
Journal of Atherosclerosis and Thrombosis	Kawabe et al.^25^	Japan/ 2014	To examine the relationship between work type and number of MS components.	MS prevalence was 6.3%; it was higher among night- shift workers (9.6%). Shift work contributed to MS when compared to daytime jobs (OR = 1.47).
Revista Latino- Americana de Enfermagem	Ribeiro et al.^26^	Brazil/ 2015	To identify the prevalence of MS among nurses, as well as its association with occupational stress, anxiety, and depression.	MS prevalence was 38.1%. Researchers identified a correlation between anxiety and MS (p = 0.022), as well as stress and SM (p = 0.008).
Acta Paulista de Enfermagem	Moreno et al.^27^	Brazil/ 2015	To verify the prevalence of MS among workers of different shifts in a metallurgical company.	A positive MS diagnosis was obtained in 26.8% of the studied population, and it was more frequent in those working from 6 a.m. to 2 p.m. (55.2%).
Revista de Saúde Pública	Canuto et al.^28^	Brazil/ 2015	To analyze if MS and its components are associated with demographic, socioeconomic, and behavioral aspects of workers on fixed shifts.	MS prevalence was 9.3%. Work shifts were not associated with the components of MS.
Revista Cubana de Medicina Militar	Sotolongo et al.^29^	Cuba/ 2015	To determine the cardiovascular risk of employees of a health care institution, according to occupation and duration of employment.	MS prevalence was 13.2%. A longer duration of employment (4 years) increased the occurrence of MS from 4.9% to 16.7%.
British Medical Journal	Lajoie et al.^30^	Canada/ 2015	To investigate the association between shift work and MS.	Shift work was associated with MS (OR = 2.29).
PLoS One	Magnavita^31^	Italy/ 2015	To evaluate the relationship between the psychological damage caused by occupational trauma and MS.	Individuals with psychological injury had a higher prevalence of MS than other workers (4.3% vs 0.9%).
PLoS One	Garbarino &Magnavita^32^	Italy/ 2015	To evaluate the association between occupational stress factors and MS.	Police officers with high stress levels had a higher risk of developing MS (RR = 2.68). Demand and effort were significant predictors of MS.
Diabetology & Metabolic Syndrome	Strauß et al.^33^	Germany/ 2016	To compare the prevalence of MS and metabolic risk between firefighters and office workers.	MS was diagnosed in 32.6% of the office workers and in 14.4% of the firefighters.
BMC Public Health	Nam et al.^34^	Korea/ 2016	To investigate the relationships between sitting time, occupation, and MS in South Korea.	The risk of MS was higher among participants that remained seated for more than 7 hours/day (OR = 1.21). Office workers presented a risk of MS twice as high as agriculture, forestry, and fishery workers (OD = 2.01).
Horizonte Médico	González-Vereau &Alfaro^35^	Peru/ 2017	To investigate a relationship between occupation and MS among employees of the public sector.	MS prevalence was 2.1%; within the administrative sector, it was 2.3%.
Asian Nursing Research	Yu^36^	Korea/ 2017	To investigate sex differences in the relationship between long working hours and MS.	Female employees working 60 hours/week had twice as many chances of having MS in comparison to those who worked between 50 and 51 hours/week (OR = 2.21).
Journal of Occupational and Environmental Medicine	Bulka et al.^37^	United States of America/ 2017	To evaluate the relationship between MS and the exposure to solvents, metals, and pesticides.	MS prevalence was 27.3%; there was no association between MS and occupational exposure.
Sleep Medicine	Itani et al.^38^	Japan/ 2017	To investigate the effects of lifestyle aspects (including hours of sleep, shift work, and off days) on MS.	MS prevalence was 16.9%. Shift work significatively promoted MS (RR = 1.06).
Medicine	Yeh et al.^39^	Taiwan/ 2018	To explore the prevalence of MS in various employee groups in a hospital in Taiwan.	The global incidence of MS was 12%. Doctors and administrative employees were at higher risk of MS than other professions.
International Journal of Occupational and Environmental Medicine	Jeong^40^	Korea/ 2018	To investigate whether the work environment is associated with MS.	MS occurrence was 19.8%. Exposure to cutting fluid was positively associated with MS (PR = 1.78).
Journal of Occupational Health	Yamaguchi et al.^41^	Japan/ 2018	To investigate the associations between work-related stressors and their alterations with a risk of MS.	Increasing changes in stressors over time presented a higher risk of MS (RR = 3.27) when compared to low work demands.
International Journal of Environmental Research and Public Health	Cho & Koo^42^	Korea/ 2018	To determine the prevalence of MS according to sex and employment type.	Non-standard workers presented a higher prevalence of MS in comparison to standard employees.
Industrial Health	Oh & Yim^43^	Korea/ 2018	To analyze the association between shift work and MS.	Two-shift rotation and MS were positively associated (RR = 1.72).
International Journal of Occupational and Environmental Medicine	Mehrdad et al.^44^	Iran/ 2018	To determine the relationship between MS and its determinants with job rank among employees of a large car factory in Iran.	MS prevalence was 7.7%; MS and job rank did not appear to be associated.

OR: odds ratio; PR: prevalence ratio; RR: relative risk; MS: metabolic syndrome.

## DISCUSSION

The profile of the selected studies pointed to English as the main language of scientific publications, since even studies conducted by Brazilian and Korean researchers or those of other nationalities were mostly published in English-language scientific journals. It is also known that North America and Europe concentrate the scientific journals with the most prestige and that are sought by researchers worldwide; the movement for the development of Asian and Latin American scientific journals is still in its early stages.

An important finding of this review is the fact that American and Asian countries accounted for most of the published studies on this subject, notably Brazil and Korea. In Brazil, the field of occupational health is being consolidated and numerous studies have been published in recent years. On the other hand, the Korean aspiration for economic and academic leading positions has required investments in research and technology and resulted in a considerable number of publications.^45^

Most of the evaluated publications were cross-sectional studies, representing a moderate level of scientific evidence.

Although it is known that cross-sectional studies are useful for determining the relationships between exposition and effect, they do not allow causal inferences between the studied variables.^46^ More robust, longitudinal studies are necessary to uncover the relationships between various occupational aspects and MS.

Medicine was the area of knowledge with the most publications, since MS is a clinical condition classically studied by this specialty. However, the considered exposure involved occupational aspects within the area of Occupational Medicine, which presented less publications. This could be related to the fact that medical journals are often considered for publication by many researchers due to their academic prestige.

Occupation was the most recurrent occupational aspect in the evaluated studies. This variable determines various exposures related to MS, such as income (which in turn determines lifestyle and access to healthy foods, as well as spaces for physical activity and leisure). Occupation also determined the level of physical activity performed during work. Regarding this aspect, the studies were not unanimous, although most of them revealed that the higher the levels of physical activity during work (such as that performed by manual workers), the lower the occurrence of MS; this is due to the energy expenditure during work. Workers that performed sedentary activities, such as supervisors and managers, presented a higher occurrence of MS due to a lower energy expenditure. In addition, these employees were under more psychological pressure at work owing to more complex work activities and more responsibility, resulting in many cases of occupational stress, which is a risk factor for MS.^44^^,^^47^^-^^49^

Shift work was also associated with the occurrence of MS. Changes in the circadian rhythm and in meal, sleep, and rest times caused by night shifts resulted in a higher occurrence of MS among the studied workers. Sleep deprivation results in insulin resistance and increased serum levels of cortisol, in addition to interfering with appetite regulation. These factors, together or isolated, contribute to the development of obesity, hypertension, and ultimately MS. Night work can also contribute to an indisposition for physical activity, which could act as a protective factor against MS.^50^

Regarding occupational stress, the triggering of a fightor-flight response derives from the exposure to stimuli in the work environment that surpasses homeostatic capacity. The recruitment of the hypothalamic-pituitaryadrenal neuroendocrine axis promotes a response with the release of cortisol by the suprarenal glands. However, a persistent and excessive release of cortisol can ultimately lead to adipogenesis, hyperglycemia, and hypertension, which are components of the metabolic syntrome.^51^

## CONCLUSIONS

In this study, a positive association of the exposure to certain occupational aspects with MS became evident throughout various occupational categories. This finding is relevant because it allows a consolidation of the published knowledge and strengthens the evidence regarding work as a disease-promoting agent.

For a long time, the concept of work-related risks was linked to physical aspects such as ergonomics, exposure to dangerous substances, noise, or temperature. Although the subjective aspects of labor are considered occupational risks since Taylorism, these were not considered by scientific studies as determinant factors for the workers’ fatigue and illness.

Currently, the field of occupational health offers numerous perspectives and possibilities of investigating problems related to the execution of work activities. This execution can determine the individuals’ way of being and doing, consequently delineating determinate profiles of morbidity and mortality among workers. In view of the central role played by work in people’s lives, the need to deeply investigate it and all aspects of its composition and characterization becomes evident.
